# Supplementary material for: Cryo-EM structure in situ reveals a molecular switch that safeguards virus against genome loss
Source: eLife. 2020 Apr 14;9:e55517. doi: 10.7554/eLife.55517 (PMC7234808; doi:10.7554/eLife.55517)
Supplement: Supplementary file 1. [file elife-55517-supp1.docx]

| **Data collection** |  | | | |
| --- | --- | --- | --- | --- |
| Microscope | Titan Krios | | | |
| High Tension / kV | 300 | | | |
| Pixel size, unbinned / Å | 1.065 | | | |
| Spherical aberration / mm | 2.7 | | | |
| Nominal magnification | 75000 | | | |
| Nominal defocus / μm | 0.5–2.5 | | | |
| Detector (mode) | Falcon 3EC (integrating) | | | |
| Accumulated dose / eÅ^-2^ | 99 | | | |
| **Refinement and model statistics** | | | | |
| Symmetry | C12 | | | |
| Resolution (FSC 0.143) | 3.74 | | | |
| Map-to-model correlation | 0.835 | | | |
| MolProbity score | 1.36 | | | |
| EMRinger score | 2.56 | | | |
| RMS deviations |  |  |  |  |
| Bond lengths / Å | 0.005 | | | |
| Bond angles / ^o^ | 0.842 | | | |
| Ramachandran plot / % |  |  |  |  |
| Favored | 94.62 | | | |
| Allowed | 5.38 | | | |
| Outlier | 0.00 | | | |
